# Correlation and Change in Physical Activity and Physical Fitness across Four Years of College Students after One Year of COVID-19 Lockdown

**DOI:** 10.3390/healthcare10091691

**Published:** 2022-09-05

**Authors:** Hongyan Yu, Shicheng An, Yiming Tao, Larry Austin

**Affiliations:** 1 Physical Education Department, Shanghai Jiao Tong University, Shanghai 200001, China; 2 International Division, Shanghai Gezhi Middle School, Shanghai 200001, China

**Keywords:** physical activity, physical fitness, college students, PE course, COVID-19, academic years

## Abstract

The relationship between physical activity (PA) and physical fitness (PF) has been well established among college students. However, the impact of this relationship after 1 year of COVID-19 pandemic lockdown measures is unclear. This study aimed to test the relationship between PA and PF, exploring the trend across four years, the different components of PF related to PA, and their determinants, by analyzing specific items. A total of 1506 university students (19.48 ± 1.35 years old, 55.8% male) in years 1–4 at two comprehensive universities in Shanghai were recruited after one year of COVID restrictions and asked to complete the PF measurements and International Physical Activity Questionnaire (IPAQ Chinese Short version). The PA level is categorized into three types of intensity (low-moderate-high), and the level of PF is represented by the total test score of each item. Results show that PA was significantly positively correlated with PF; PA levels significantly predicted 1000m-run, 50m-sprint, and standing-long-jump in males, and 800m-sprint and sit-ups in females. Males predominantly had high-intensity PA, whereas females maintained moderate-intensity PA over four academic years. Meanwhile, PA and PF both trended downward as academic years increased in males and females, which could be attributed to a decline in high-intensity PA. The following two recommendations were obtained from the study: first, college students should engage in high-intensity PA activities after the end of the epidemic. Second, colleges offer physical education classes for four academic years of college students to promote PA and PF.

## 1. Introduction

The COVID-19 pandemic has led to several impairments in physical and mental health [1]. It presents an unprecedented challenge to public health (WHO, 2020). Following the detection of COVID-19 in Wuhan at the end of 2019, the Chinese government implemented successive restrictive measures to block the virus’ spread by close contact. During this period, schools, gyms, and commercial centers, among others, were closed; all school teaching activities were changed to online learning at home, and everyone was asked not to leave their homes unless necessary.

Two studies reviewed have shown that the direct impact of lockdown measures resulted in a decrease in PA [2,3], which in turn leads to many psychological issues, such as increased feelings of anxiety, stress, and depression [4], and sleep disorders [5], as well as decreased immune defense [6], physical fitness level [7], mental well-being [8], and cognitive abilities [9]. The health benefits of physical fitness (PF) and physical activity (PA) have been reasonably established [10]; however, college students are especially vulnerable to changes in psychology and physiology. One study found that college or university time is a critical period regarding unhealthy changes in energy-related behaviors in students [11], more than 80% of adults continue the same PA patterns that they established during their college [12], and low PF in college is proven to track into adulthood [13], some studies found COVID-19 even changed exercise habits among college students [14], so the restriction post-impact on the PA and PF of college students may be more severe than expected.

Previous studies have confirmed that PA and PF are closely related to college students, and they both tend to decrease with the academic year. For example, a study with 17-year-old girls in Lithuania showed a close association between PA and PF [15]. A survey of PA and PF among college students from eight Asian cities found that those who could meet PA criteria were also able to meet the Healthy Fitness Zone (HFZ) in terms of aerobic capacity and muscle strength [16]. Small et al. showed that Belgian university students’ daily PA declined significantly from the first to the seventh semester [17], and Ode et al. maintained that the decrease occurred even before and after two semesters of the same year [18]. Another study assessing PA using an accelerometer found that average, moderate, and vigorous-intensity PA was lower with higher age in both sexes [19]. Meanwhile, some studies have interpreted that PF levels during college usually decrease during academic years [20,21,22,23], and the significant changes include a reduction in the percentage of students in the maintenance phase for aerobic exercise participation and a corresponding decrease in the contemplation stage for stretching [24]. It revealed that college students’ PA and PF have decreased, and they are unlikely to improve as students get older.

After prolonged COVID-19 restrictions, college students returned to campus, and their PA comes primarily from daily life activities on campus, including physical education classes and extracurricular sports activities. During the restriction period, the PA of college students was mainly from daily life at home. On the other hand, to date, most of the studies on the effects of the related COVID-19 pandemic on PA and PF have focused on the onset of the epidemic, but the relationship between PA and PF after one year of lockdown remains unclear. Thus, the objectives of this study were as follows: (1) to explore the relationship between PA and PF of students in four years of college after one year of lockdown restriction, and (2) to determine the effect of PA levels on PF and PF components. By elaborating on the relationship between PA and PF in this context, we expect the results of this study to provide a reliable reference for college PE class settings.

## 2. Materials and Methods

### 2.1. Study Design and Sample

This is a cross-sectional study with a random sample. We recruited participants from the following two universities: Shanghai Jiao Tong University and Tongji University. They are public, comprehensive universities located in Shanghai, and students come from all over the country, making the samples more universal and representative.

During the weekend of November-December 2020, the Universities conducted the National Student Physical Fitness Test. During the test, students were free to choose the date and time to take the test. We recruited participants from all college students who came to the test. The inclusion criteria for the college students included (1) over the age of 18; (2) healthy (no chronic diseases, no history of stroke, myocardial infarction, diabetes, malignancy, etc.); (3) those who return to school to participate in normal learning activities after experiencing the restrictions of the mainland COVID19 in 2020; (4) availability to complete the National Student Physical Fitness Test (NSFT) and the International Physical Activity Questionnaire (IPAQ).

Online sample size calculator was used to obtain sufficient statistical power [25]. According to the data of the Shanghai Bureau of Statistics, Shanghai has 526,300 college students [26], so the sample size of no less than 1500 was calculated using a calculator based on 526,300 college students, a 3% margin of error, a 95% confidence level, and a 50% response distribution.

### 2.2. Data Collection Procedures and Tools

Participants were required to complete the National Student Physical Fitness Test and IPAQ. The National Student Physical Fitness Test was divided into an indoor test and an outdoor test. After participants completed the questionnaire, they started completing the indoor items and then completed the outdoor items the next day. These two tests were conducted between October 2020 and December 2020, 1 year after the pandemic lockdown.

A professional online questionnaire survey tool named “Questionnaire Star” was used for data collection. Prior to the testing, a comprehensive verbal description of the nature and purpose of the test and its experimental risks was elaborated to each participant. At the same time, participants were informed that they could withdraw freely without any reason. Students who indicated their willingness to participate were instructed to scan the code with their cell phones. The QR code was generated on “Questionnaire Star”. After the code was scanned, the content of the participant’s informed consent was displayed on the cell phone screen. If participants read it and selected “I agree”, they were considered voluntarily enrolled and well informed of the purpose and risks of the test, as well as they agreed with the use of the test data for the research.

At the end of each week’s testing, we eliminated invalid samples, including unreasonable filling, regularity, continuity in answering, etc. The procedure was repeated during the testing period until the estimated sample size was reached. Finally, 1506 participants (19.48 ± 1.35 years old) were from grades one to four, including 840 males (19.47 ± 1.40) and 666 females (19.49 ± 1.29).

### 2.3. Physical Fitness Measurement

Physical fitness measurements were based on the National Student Physical Fitness Test (NSPFT). NSPFT is a battery of tests issued by the Ministry of Education of the People’s Republic of China. It is currently a government-designated test used to evaluate the physical fitness level of university students in China [27]. The testing team consists of experienced physical education teachers and graduate students in kinesiology, as well as test quality supervisors. Testers must pass rigorous testing training. Trained testers conducted height, weight, sit-and-reach, and vital capacity measurements indoors. PE teachers conducted field-based physical fitness measurements outdoors, including 50m-sprint, standing, long jump, pull-up (male)/sit-up (female), and 1000m-run (male)/800m-run (female) (Table 1). To reduce measurement bias, counting item scores were judged by 2 testers together. For the running items’ time recording, three testers used an electronic stopwatch to record the completion time. The second-best time was recorded as the final score. The 1000m/800m run is accurate to 1.0 s. The 50m run is accurate to 0.1 s. The procedures and devices used for the tests were in accordance with the equipment standards of the NSFT (revised in 2014). The total PF score was the sum of each PF component score calculated according to the NSPFT Standards [27].

### 2.4. Physical Activity Measurement

To measure the individual’s PA, we used the International Physical Activity Questionnaire (IPAQ), which has both a short and a long form. The IPAQ is recommended for use in adult PA surveys in several countries, which is considered a reliable and effective way to measure PA [28].

In this study, we adopted the short version to measure the PA level of the participants. We asked participants to fill different levels of physical activity and how much time they spent each week performing specific activities, such as heavy lifting, digging, aerobic exercise, or fast cycling. Craig divided the IPAQ results into three levels (low-intensity, moderate-intensity, and high-intensity) by integrating data and calculating the number of PA types (Table 2, Table 3). Total physical activity MET-minutes/week = sum of Walking + Moderate + Vigorous MET-minutes/week scores [29].

### 2.5. Statistics and Analysis

Data were stratified by gender and academic year. Descriptive statistics showed the characteristics of participants using means, standard deviations (±SD), and percentages. The normal distributions of variables were verified through histograms and by the Kolmogorov–Smirnoff test. Two-tailed independent sample t-tests were used for normally distributed variables and Mann–Whitney’s U for non-normally distributed variables. Correlations between variables were determined using Pearson correlation analysis. A one-way analysis of variance (ANOVA) was used to compare the differences in PA (MET) and PF scores between the four academic years. A stepwise method of multiple linear regression was performed to test the influence factors between PA levels and PF scores. The univariate linear regression analysis was performed to identify the predictive relationship of PA levels on PF components. The collected data were double entered and analyzed using the IBM^®^ Statistical Package for Social Sciences version 25.0 (SPSS, Inc., Chicago, IL, USA) software. The significance level was considered at *p* < 0.05.

## 3. Results

Table 4 displays the basic characteristics of the three PA levels (low PA-moderate and PA-high PA) and PF-related variables, stratified by gender and academic year, are displayed in Table 4. There were significant differences in PA and PF scores between grades for both male and female students (*p* < 0.05). Overall, the PA or PF was different for students in each of the four academic years.

### 3.1. Physical Activity and Physical Fitness in Four Academic Years

**Figure 1:**
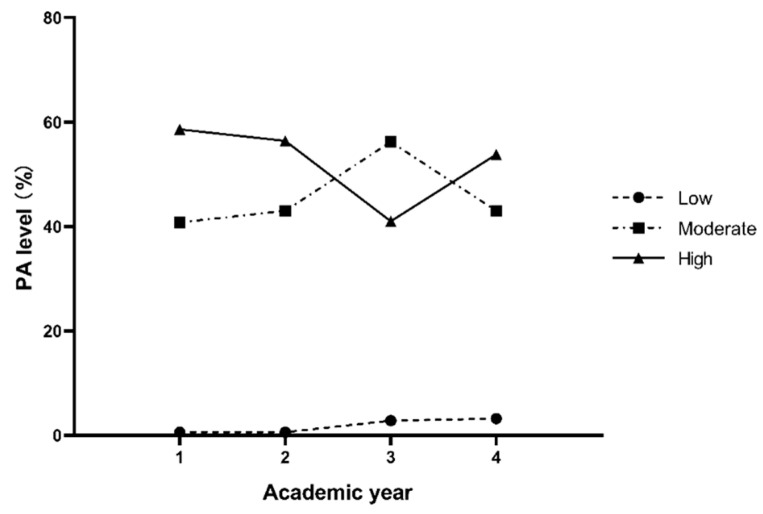
The percentages of the three levels of PA in each academic year for males.

**Figure 2:**
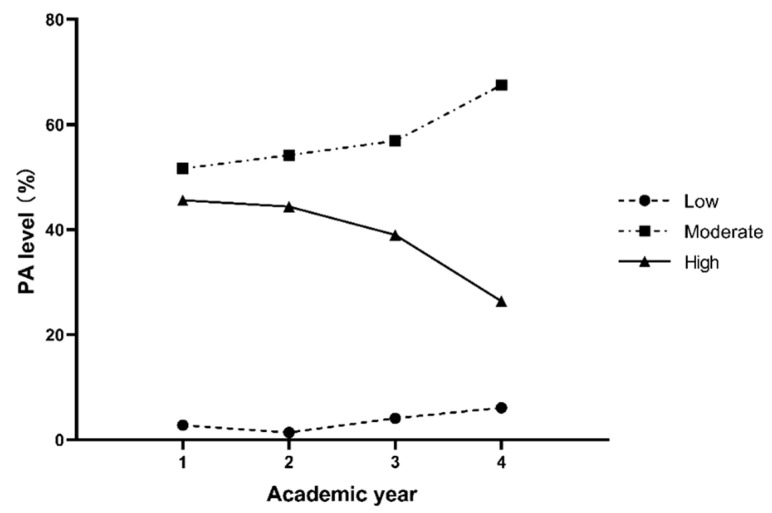
The percentages of the three levels of PA in each academic year for females.

Figure 1 and Figure 2 show the percentages of different PA levels across four years in males and females, respectively. The correlation between the PA level and academic year in males (r = −0.099, *p* = 0.004) and females (r = −0.135, *p* < 0.001) was negative. Both males and females had predominantly moderate PA or high PA in all four years. The percentages of moderate and high PA for males reached 45.8% and 52.5%, and 57.6% and 38.9% for females, respectively, while the percentages of low PA for males and females were only 1.8% and 3.6%.

**Figure 3:**
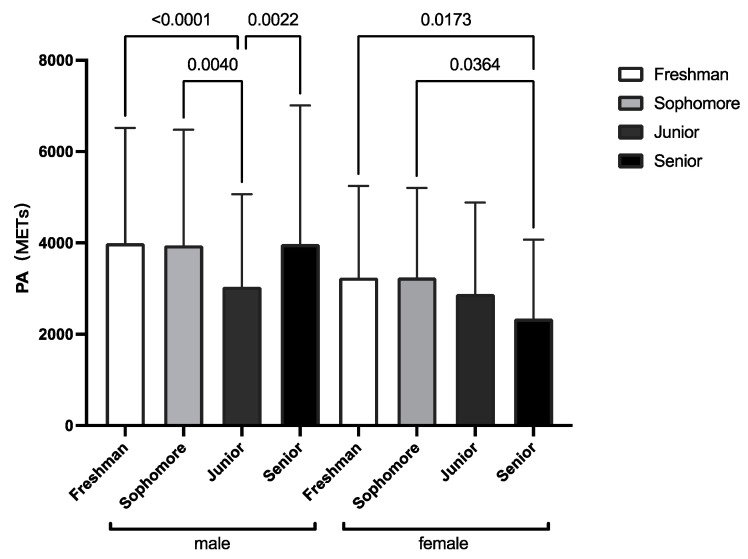
The PA (METs) in different academic years in males and females.

Figure 3 shows the comparison of male and female PA (METs) over the four academic years. In particular, the freshman and sophomore PA (METs) were higher than those of juniors and seniors, and some of the differences were significant.

**Figure 4:**
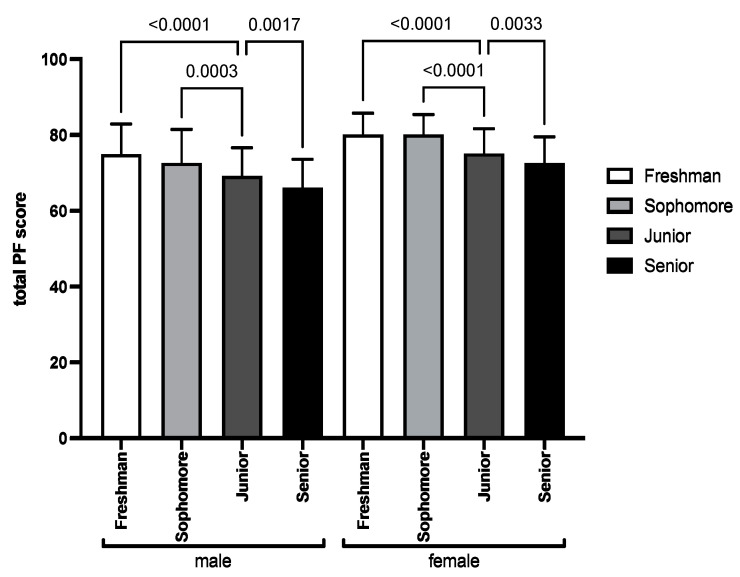
The total PF score in different academic years in males and females.

Figure 4 shows the mean and standard deviation of the total PF score for males and females in the four academic years. The correlation between total PF score and academic year for males (r = −0.392, *p* < 0.001) and females (r = −0.441, *p* < 0.001) was significantly negative. Both freshmen and sophomores had significant differences with juniors in total PF scores in both sexes.

### 3.2. Relationship between Physical Activity and Physical Fitness and Physical Fitness Components in Four Academic Years

**Figure 5:**
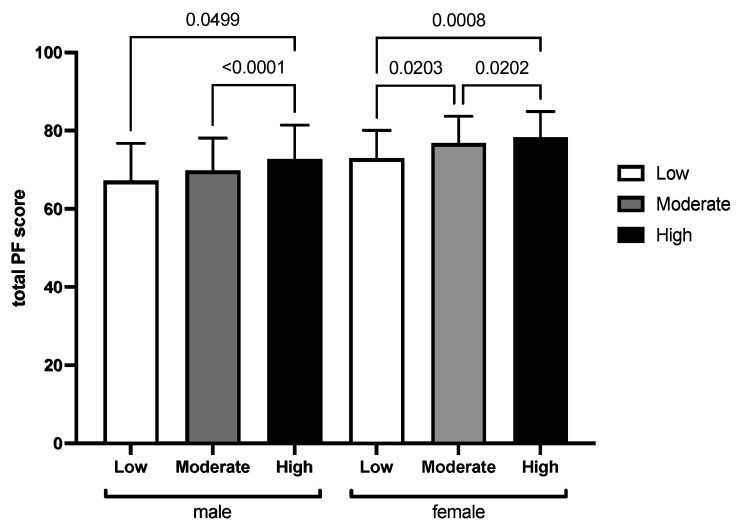
Mean and standard deviation of PF at different PA levels in males and females.

Figure 5 shows the mean and standard deviation of the total PF score at different PA levels in males and females. From the column diagrams, we discovered a significant linear correlation between PA levels and total PF score in both males (r = 0.163, *p* < 0.001) and females (r = 0.132, *p* < 0.001). Therefore, the higher the PA level, the higher the PF scores for both sexes. The multiple linear regression results showed that the regression equation was significant (F = 189.527, *p* < 0.001), where both gender (b = 6.392, β = 0.378, *p* < 0.001) and grade (b = −2.712, β = −0.359, *p* < 0.001) affected the total PF score. Age did not affect the total PF score (β = 0.076, *p* = 0.052). These variables explained 27.3% of the variance in the total PF score.

**Figure 6:**
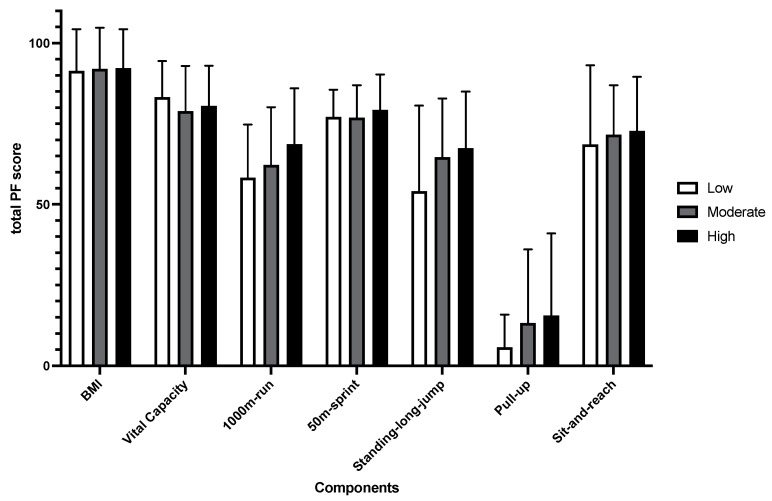
Mean and standard deviation of PF components at different PA levels in males.

**Figure 7:**
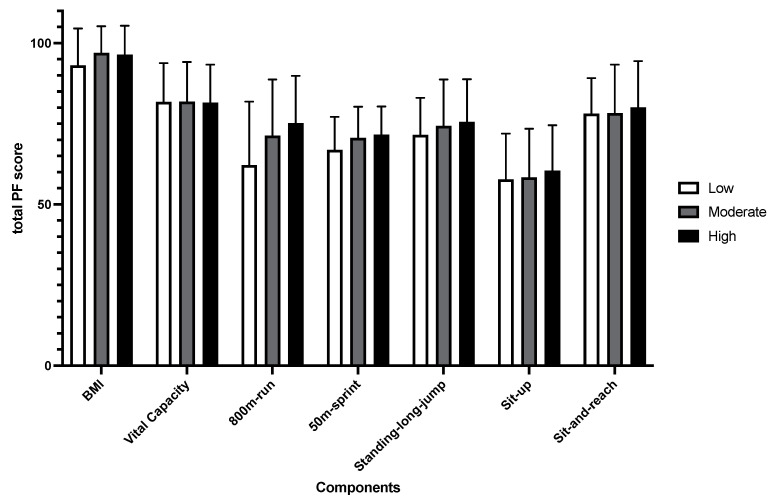
Mean and standard deviation of PF components at different PA levels in females.

Figure 6 and Figure 7 show the mean and standard deviation of PF components at different PA levels and the correlation between the PA level and PF components of males and females, respectively. The results of linear regression of each PF component with PA levels showed that the performance of male’s 1000m run (b = −12.490, β = −0.185, *p* < 0.001), 50m sprint (b = −0.120, β = −0.085, *p* < 0.05), and standing-long-jump (b = 4.412, β = 0.110, *p* < 0.05) could be significantly predicted by PA levels. For females, it was the 800m run (b = −0.002, β = −0.112, *p* < 0.05) and sit-up (b = 0.008, β = 0.090, *p* < 0.05). No other components were significantly predicted.

## 4. Discussion

This study aimed to verify the relationship between PA and PF within academic years and determine whether different PA levels could cause differences in PF and PF components after 1 year of restriction. The results revealed a significant linear correlation between PA and PF in both sexes, and both PA and PF decreased each academic year. This result is consistent with other studies [18,20,21,22,23,30,31]. Several studies have found that the COVID-19 outbreak increased college students’ intuitive exercises and more positive attitudes [14], and positive self-perception and motivation for fitness exercises during the pandemic [32]. However, from the results of the study, the trend of PA and PF among college students was consistent with the previous findings. This may be due to the so-called “gap” between intention to exercise and actual participation in recommendations and policies that should facilitate and strengthen PA and PF for college students even after the COVID-19 outbreak.

### 4.1. Physical Activity Level in the Four Academic Years

In our study, females consistently had the highest percentage of moderate PA in the four years, and males had the biggest percentage of high PA in all but the junior year. Meanwhile, the percentage of low PA is the lowest each year for both sexes but increases with each academic year. The PA scale used in the study can be repetitive, structured, planned (e.g., a fitness class or recreational activities such as hiking), leisurely (e.g., gardening), sports-focused (e.g., basketball, volleyball), work-related (e.g., lifting and moving boxes), or transportation-related (e.g., walking to school) [33]. We are all convinced that PA is significantly influenced by the living environment [34]. The campus, where the participants were from in this study, is 309.25 hectares (4638.75 acres) and has 94 dormitories with a total building area of 309,000 square meters. There are hospitals, post offices, banks, theaters, student activity centers, various sports venues, and other facilities on campus, forming a relatively independent community. Shared bicycles are provided everywhere on campus. Cycling is the main mode of transportation for students, and students ride bicycles between the teaching blocks daily. In the questionnaire, participants were asked, “Over the past 7 days, how many days were you physically in vigorous PA, such as fast cycling?” There was no significant difference in this question for the fourth academic year students. Therefore, we speculate that the resumption of normal campus life is the likelihood that most college students will maintain their PA even after the end of restrictions.

### 4.2. Physical Fitness in the Four Academic Years

Another point worth noting is that the transition from sophomore to junior may become an important demarcation for PA and PF levels. Chinese college students are regarded as a special group on this issue. The study on students at Tsing Hua University also discovered a decline in both PA and PF of Chinese college students with an increase in years, and the total scores of PF increased from year one to grade two, then gradually slowed from year three to year four [35,36]. In China’s higher education system, freshmen and sophomores are required to complete certain physical education credits, but not juniors and seniors. According to the Basic Standards of Physical Education in Colleges and Universities [37], freshmen and sophomores are asked to participate in two PE classes, 45 min each, once a week. At the same time, a quality exercise program with tests reflecting the cardiopulmonary function of students should be implemented in the PE class. Moreover, the students in the PE class were stipulated to run at least 2 km after school every day, and it counted as 10 points in the PE class score. In addition, each semester of the first two years of university, they are required to take the NSPFT test, which accounts for 35% of their PE class grades. All of these were included in the individual’s grade point average (GPA), but juniors and seniors are not required to take PE classes, which may be the main difference between grades 1/2 and 3/4. Although the correlation between the PE class and PA or PF was obvious, considering the special situation of Chinese college students, we are convinced that the PE class helped students maintain PA levels [38] and health-related fitness [39], and the benefits of taking PE classes go beyond that. The PA in the PE class is a typical sportive PA, not work-related or housework-related PA. A study indicated that both genders showed cognitive benefits from greater sportive PA but not from non-sportive PA [40]. PE classes can be helpful for inactive college students. Some scholars have proposed that there is a strong need for more active PE classes for college students to develop PF and unsatisfactory PA [12,41], so we believe that taking PE classes to reduce the impact of restrictions on PA and PF is a recommended method.

### 4.3. Correlation between Physical Activity and Physical Fitness

Our results revealed that there is a significant linear correlation between PA and PF levels in both sexes. The sums of the percentages of moderate and high PA for males and females were 98.2% and 96.4%, respectively. Meanwhile, the percentage of low and moderate PA increased with the academic year, while the percentage of high PA decreased from year to year. Therefore, the decline in PF for both sexes is due to the decrease in high PA. It can be seen that the effect of high PA on PF is important. A study found that 17-year-old girls with high PA levels perform better in the standing-long-jump and have a lower BMI than others [15]. Leppänen, M.H., et al. confirmed that time spent on vigorous PA is associated with higher fat-free BMI and better physical fitness among college-age students [42]. Osipov et al. showed that college students with higher levels of PA were better at the PF compared to those with lower levels of PA [43]. Similarly, Ruiz et al. demonstrated that regular participation in moderate and vigorous exercise could increase PF, especially sportive PA, leading to many health benefits [44]. Moreover, a study found that university students with a high level of PA scored 2.39 times better on the strength test and 1.39 times better on the long jump test [35]. Even vigorous intensity activities for university students may provide improved mental health [45,46,47,48]. However, a study illustrates that adding specific required PA components does not significantly improve a college student’s attitudes and perceptions toward their personal health [49]. Even some vigorous PA, such as part-time jobs and extracurricular sports clubs, could cause unexpected negative side effects (such as depressive tendencies [50]), fatigue and confusion [51]. A systematic review conducted by Maselli M. et al. suggested that PA promotion interventions should address a range of behavioral determinants [52]. Therefore, it could be surmised from this study and similar studies that high PA is beneficial to college students, but the choice of activity is an important factor in determining its effectiveness.

The results showed that PA also affected specific PF components. According to the correlation results of PA and PF component scores, the 1000m-run, 50m-sprint, and standing-long-jump for males, and 800m-run and sit-up for females were significant. In PF measurements, these items were adopted to test the speed, endurance, and strength of individuals, including trunk strength and explosive strength. This finding gives us a new understanding of the specific items of PF affected by PA in university students. Murphy et al. identified that using PA to improve aerobic fitness appears to be the most effective at maintaining healthy body composition in college-age women [53]. A study that objectively measured PA found a moderate association between lower limb muscle size and muscle strength [54]. Similarly, PA can also strengthen the vertebrae and discs, which is an essential requirement as weak spinal strength is becoming a severe PF issue in teenagers and adults [55]. Nevertheless, a study discovered that PA is not related to flexibility in 17-year-old girls. In a study discovered [15], the same result was also found in our study, and health and PA professionals in higher education have not been able to effectively increase students’ PA behaviors [56]. Such results may be helpful for PE teachers and students in university to improve PF by PA after the restriction.

### 4.4. Limitations and Implications

This study has the following limitations: (1) This study uses a cross-sectional research design, so the results could not infer the causal relationship between variables. (2) PA was measured using a self-reported questionnaire. Therefore, it is susceptible to potential recall bias, unreliable estimates, and misinterpretation of questions. (3) We only examined the effects of gender, grade, and age on the relationship between PA and PF. In reality, their relationship is likely to be influenced by other factors as well, so the findings may be more complex than the present study shows. A follow-up study design could be used in the future to validate the findings of this study.

Despite these limitations, the study has obvious practical significance. To our knowledge, this is the first study exploring the relationship between PA and PF after one year of COVID-19 restriction. The results indicate that PA and PF still tend to decline with the increased academic year and that increasing high PA to improve speed, endurance, and strength becomes a goal after 1 year of lockdown. This contributes to targeted policies or recommendations facilitated for college students after the pandemic outbreak.

## 5. Conclusions

This study demonstrated a significant correlation between PA levels and PF in college students. Both male and female students showed a decreasing trend in PA and PF with the increasing academic year. In addition, speed, endurance, and strength were strongly correlated with PA levels, and the decline in high PA led to a decline in PA and PF following 1 year of COVID-19 restrictions.

## Figures and Tables

**Table 1:** National Student Physical Fitness Standard Test items and scoring weights.

Components	Score Weight(%)
Height (cm)	15
Weight (kg)	
Vital capacity (mL)	15
50m-sprint (s)	20
Sit-and-reach (cm)	10
Standing long jump (m)	10
Pull-up (male) (r)	10
Sit-up (female) (r/min)	
1000m-run (male) (s)	20
800m-run (female) (s)	
Total score	100

**Table 2:** Formula for calculating metabolic equivalents for computation of MET minutes.

Level (MET-Minutes/Week)	Physical Activity	Calculation Formulas
Low intensity	Walking	3.3 × walking minutes × walking days
Moderate intensity	Lifting light objects, cycling at normal speed or playing tennis in pairs, etc.	4.0 × moderate-intensity activity minutes × moderate days
High intensity	Lift heavy objects, use equipment for digging, high-intensity aerobic exercise or fast cycling, etc.	8.0 × high-intensity activity minutes × vigorous-intensity days

**Table 3:** International Physical Activity Questionnaire (short form) category criteria.

PA Levels	Category Criteria
High intensity	High-intensity PA > 3 days and total PA ≥ 1500 MET-min/week OR Any combination of walking, moderate-intensity ≥ 7 days or total high-intensity PA ≥ 3000 MET-min/week
Moderate intensity	High-intensity PA of at least 20 min per day ≥3 days OR Moderate-intensity PA and/or walking of at least 30 min per day ≥5 days OR Any combination of Low-, moderate-intensity PA ≥ 5 days or high-intensity PA ≥ 600 MET-min/week
Low intensity	No activity was reported OR Some activities were reported, but they do not yet meet the above criteria for moderate- and high-groups level

**Table 4:** Descriptive Statistics of Physical Activity and Physical Fitness in each academic year.

	Freshman		Sophomore		Junior		Senior	
	Male (309)	Female (213)	Male (156)	Female (144)	Male (217)	Female (195)	Male (158)	Female (114)
Age	18.19 ± 0.73	18.21 ± 0.72	19.20 ± 0.86	19.13 ± 0.58	20.22 ± 0.82	20.20 ± 0.74	21.21 ± 0.80	21.12 ± 0.67
Height/cm	173.39 ± 5.88	161.91 ± 5.68	173.69 ± 6.32	162.44 ± 5.67	173.70 ± 6.91	161.81 ± 6.26	174.47 ± 6.50	162.74 ± 5.12
Weight/kg	66.55 ± 11.14	54.51 ± 7.33	67.25 ± 12.31	53.75 ± 7.15	67.88 ± 11.93	55.02 ± 8.25	69.14 ± 12.33	55.73 ± 8.28
BMI	22.13 ± 3.46	20.80 ± 2.64	22.25 ± 3.61	20.35 ± 2.43	22.45 ± 3.29	21.00 ± 2.81	22.70 ± 3.82	21.01 ± 2.78
Vital Capacity/mL	4391.9 ± 638.2	3128.1 ± 456.7	4434.4 ± 694.4	3027.7 ± 458.1	4513.0 ± 689.5	3108.6 ± 492.9	4494.2 ± 789.4	3129.9 ± 508.7
1000 m (800 m) run/s	231.9 ± 25.6	223.2 ± 25.6	245.5 ± 31.3	227.7 ± 20.8	261.6 ± 35.2	246.7 ± 39.6	276.1 ± 37.6	256.4 ± 42.4
50 m run/s	7.16 ± 0.57	8.82 ± 0.59	7.37 ± 0.59	8.79 ± 0.55	7.53 ± 1.07	9.19 ± 1.01	7.63 ± 0.58	9.38 ± 0.66
Standing Long Jump/cm	236.34 ± 21.89	180.70 ± 15.81	231.64 ± 22.61	183.89 ± 16.31	226.13 ± 18.64	173.12 ± 16.41	219.47 ± 17.75	168.13 ± 17.23
Pull-up (Sit-up)	4.91 ± 4.17	30.75 ± 6.27	5.26 ± 4.57	32.61 ± 6.37	5.03 ± 4.17	30.23 ± 6.46	3.96 ± 3.89	29.28 ± 6.42
Sit and Reach/cm	14.26 ± 7.12	19.05 ± 7.08	13.92 ± 7.25	19.08 ± 5.89	13.97 ± 6.69	18.10 ± 6.25	13.24 ± 6.49	18.78 ± 5.80
PF total Score	74.84 ± 8.08	80.01 ± 5.74	72.57 ± 8.94	80.09 ± 5.32	69.11 ± 7.55	75.05 ± 6.63	66.06 ± 7.54	72.55 ± 6.98
*PA (METs)*	3992.68	3036.81	3975.97	3944.78	3236.23	2890.76	2341.77	3241.50
*PA (%)*								
Low	0.6	2.8	0.6	1.4	2.8	4.1	3.2	6.1
Moderate	40.8	51.6	43.0	54.2	56.2	56.9	43.0	67.5
High	58.6	45.6	56.4	44.4	41.0	39.0	53.8	26.4

Note. Continuous variables are shown as s mean ± standard deviation (M ± SD), PA are shown as median METs–minutes/week volumes and percentage at each PA level (%); Abbreviations: PA, physical activity; PF, physical fitness; BMI, body mass index; PF Score, the sum of each PF component score, the full score is 100. 1000m run is for males, 800m run is for females; pull-up is for males, sit-up is for females.

## Data Availability

The datasets used and/or analyzed during the current study are available from the corresponding author on reasonable request.
